# Innovating From Within: A Process Model for User-Centered Digital Development in Academic Medical Centers

**DOI:** 10.2196/11048

**Published:** 2018-12-19

**Authors:** Sara Kuppin Chokshi, Devin M Mann

**Affiliations:** 1 Department of Population Health New York University School of Medicine New York, NY United States; 2 New York Univeristy Langone Health Medical Center Information Technology New York, NY United States

**Keywords:** academic medical centers, digital health, heath information technology, innovation, process model, user-centered design

## Abstract

**Background:**

Design thinking and human-centered design approaches have become increasingly common in health care literature, particularly in relation to health information technology (HIT), as a pathway toward the development of usable, diffusible tools and processes. There is a need in academic medical centers tasked with digital innovation for a comprehensive process model to guide development that incorporates current industry trends, including design thinking and lean and agile approaches to digital development.

**Objective:**

This study aims to describe the foundations and phases of our model for user-centered HIT development.

**Methods:**

Based on our experience, we established an integrated approach and rigorous process for HIT development that leverages design thinking and lean and agile strategies in a pragmatic way while preserving methodological integrity in support of academic research goals.

**Results:**

A four-phased pragmatic process model was developed for user-centered digital development in HIT.

**Conclusions:**

The model for user-centered HIT development that we developed is the culmination of diverse innovation projects and represents a multiphased, high-fidelity process for making more creative, flexible, efficient, and effective tools. This model is a critical step in building a rigorous approach to HIT design that incorporates a multidisciplinary, pragmatic perspective combined with academic research practices and state-of-the-art approaches to digital product development to meet the unique needs of health care.

## Introduction

### Background

User-centered design (UCD) has been applied in the development and testing of software and technology for decades; however, the application of UCD and design thinking in health care innovation and health information technology (HIT) is a more recent phenomenon [1-3]. Given that the field of UCD in HIT is relatively nascent, albeit increasingly common, a comprehensive process model is yet to be established for applying this approach and its associated methodologies to the design of digital tools for health care delivery. In this paper, we propose an integrated and pragmatic process model for the development and testing of HIT based on our experience using a rapid cycle, iterative, user-centered approach to the development and implementation of various types of innovations for health care research and clinical delivery. Pulling from relevant academic disciplines, as well as industries outside of health care, we propose an integrated model for HIT development and implementation that incorporates and builds upon popular trends in innovation today, offering a multiphased, comprehensive, best practices in a research-based approach to digital development in health care.

### Innovation in Academic Medicine

Innovation has become a priority in many academic medical centers with leaders in health services delivery calling for increased innovation and experimentation within their organizations through new research and operational processes that are more nimble, lightweight, and iterative than the typical processes in traditional academic medicine [4-7]. Although HIT innovation has lagged, software development and other design-related industries outside of health care have incorporated strategic design processes for more than a decade, combining major elements of design thinking, lean startup, and agile development principles [8,9]. These user-centered approaches are compatible with an increasingly patient-centered health system in which the goals of development are tools and processes that work for the humans who will use them, including physicians, other types of providers, staff, as well as patients and their families [10-13].

### Academic Goals and Industry Demands

Design thinking and UCD approaches, in general, have become increasingly common in scientific literature, particularly in relation to HIT, as a pathway toward the development of usable, diffusible tools, and processes [1,14,15]. Researchers in population health, as well as the computer, information, and design sciences related to HIT, have proposed models for incorporating user- or human-centered approaches and agile methods into technology development [10,16-20]. What is missing from these models, however, is the capacity to inform a variety of HIT development projects beyond mobile health and behavior change apps. In addition, other models lack the necessary specificity in approach and methods to be useful to research and operations teams at the forefront of building and implementing a wide variety of digital tools for patients, as well as clinicians and other staff in their health systems.

While design thinking and user-centricity as concepts are born out of the industry, they are relatively new concepts to academic health care [1,15]. Core tenets, such as the centrality of the user journey and the concept of “empathy,” have a rich history as cornerstone ideas in social science literature [21-23]. Anthropologists have been conducting ethnographic research on health and illness since the inception of the discipline; deep understanding of the social and organizational features of work and roles, particularly in medicine, has long been an object of the sociological imagination. It is the design thinking movement [24], however, which has pragmatized and popularized these social science research practices, lending them to wider use within scientific circles, including HIT development [22].

From the perspective of an academic health institution, any digital development process must consider the need to balance tensions between demands of HIT product development and our academic goal to contribute to the evidence-base supporting high-quality health care delivery through, for example, rigorous usability evaluation and related documentation [25]. To fulfill the potential of technology to markedly impact the quality of health services, our process of HIT design and development integrates foundational principles and strategies from the software development industry and applies them at the appropriate time while adapting them to the complexities of health care roles and workflows with rigorous user testing [26-31].

### Development of a Robust Process for Digital Innovation

Charged with establishing a pipeline for identifying and supporting innovative research and operations projects-related digital development at our institution, our group, consisting of both research and HIT innovators, created the medical center’s first lab expressly designed to support our institution’s researchers and clinicians in these types of efforts [32]. Our experience in this first year of the lab has revealed the importance of implementing a process for identifying, selecting, specifying, and supporting HIT projects at all stages. Throughout all of our projects, thus far, we have developed, employed, and refined our approach, process, and practices [33-35].

### Innovating From the Inside Out

Our experience and resulting model reflect our belief in the importance of building innovation internally, acknowledging that those most likely to identify with the motivations and experiences of our users—those providing and seeking care at our institution—are, in fact, within, rather than outside of our organization. Innovation supported from within leverages the valuable “pracademic” lens—a perspective that lies at the intersection of medical practice, health care delivery, and academia. Innovation work done “in-house” is more likely to be adopted and diffused within an organization, as it is the end users themselves building and refining the tools that impact their daily work [36]. While it is common for academic medical centers to bring in external consultants, a robust internal innovation team has the potential to transform an institution’s culture, spurring greater interest in innovation, as well as institutional capacity, to support it in a more efficient, sustainable way [32]. Given the complexity of health care organizations and HIT tools, those within the institution have the institutional knowledge essential to successful innovation—a lens not easily captured by outside consultants.

This paper aims to describe the components of our resulting model, reflecting our experience establishing the internal innovation capacity that supports our medical center’s academic goals with methodological integrity and rigor, while leveraging strengths and methodologies from current trends in software development and product management (design thinking, lean, and agile development) and adapting them for efficient, sustainable, user-centered HIT development.

## Methods

**Figure 1:**
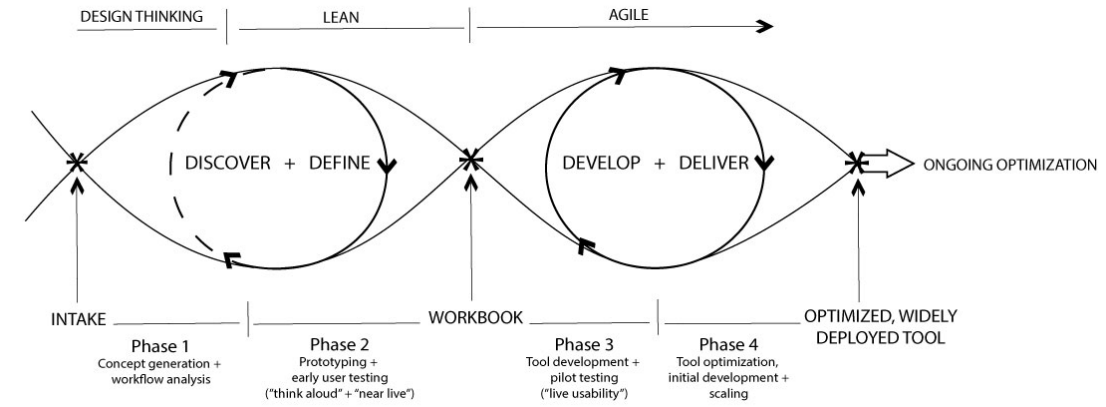
Process model for user-centered digital development.

Our integrated process model for user-centered HIT development, as seen in Figure 1, is a comprehensive picture of the entire development and testing process from concept generation to widespread deployment of an optimized tool.

Leveraging applied qualitative methods, this model incorporates the popular “double diamond” representation of the design process [9], including state-of-the-art software development strategies, a phased approach to workflow analysis, usability testing, and optimization and implementation. Tangible milestones and products are noted from the intake of a new project to ongoing optimization of the HIT tool.

## Results

### Principal Results

We used applied design thinking strategies in the predeployment phases. In phase 1 we “discovered” concept generation and workflow analysis, followed by the further definition of the problem and target of the proposed solution. Solution ideas are refined with user-testing feedback and developed throughout the lean-inspired phase 2. An agile approach, including “sprints” to tool development and delivery, occurs throughout phases 3 and 4. The binned approach to development that agile brings is key to the success of our model; however, the specifics of the sprint are beyond the scope of this paper.

In sum, our process consists of 4 phases as follows: (1) tool concept generation and workflow analysis; (2) prototyping with early user testing (including “think-aloud” and “near-live” methodologies) and iterative tool refinement; (3) tool development and pilot testing (including “live usability”); and (4) tool optimization, release, and scaling. Phases 1-3 are related to the predeployment tool design, development, workflow integration, and pilot testing, whereas phase 4 occurs after tool deployment.

### Phase 1: Concept Generation and Workflow Analysis

#### Overview

The concept generation phase features the design thinking or discovery piece of the model. The initial concept generation phase comprises the data gathering, analyses, and vetting necessary to build an initial prototype. Beginning with the very first “intake” meeting between the internal innovation and project teams, work in the concept generation phase is geared toward establishing the basic parameters of the tool to specify a minimum viable product draft of the tool. This tool will be used for the initial round of user testing with the assumption that marked iteration will occur in later phases of the process. Components of this phase include the following: extensive literature review and competitive landscape analysis of similar and related digital products on or coming to market; key informant interviews along with implementation site observation (often culminating in a design workshop aimed at producing a detailed feature list); and workflow analysis to inform phase 2 building of the initial tool “minimum viable product” prototype, as well as an initial backlog of features the project team deems as valuable but not key for the initial tool version.

#### Literature Review and Competitive Landscape Analysis

As with typical research endeavors, a comprehensive literature review occurs early in the process to establish the evidence-base necessary to understand what the current state of the technology in the field is, confirm gaps and use cases the tool could potentially address, and begin to identify where the opportunity exists for innovation for the tool in development. In addition, digital development projects benefit from a competitive landscape analysis, a review of similar or relevant digital products currently available or in development. The competitive analysis is essential to determining that the tool in development adds value by building upon rather than duplicating the contributions of those already available. Furthermore, it is a necessary first step in determining potential partners for codevelopment, should the development project be compatible with such an approach.

#### Key Stakeholder Interviews

Concurrent with the literature review and competitive analysis, interviews with key stakeholders are critical in identifying “pain points” (key needs the tool might address), identifying real-world workflow issues (and resulting opportunities for the tool to intervene or facilitate), and confirming potential use cases as identified in the literature. Individual semistructured interviews with key stakeholders typically last 60-90 minutes and are structured to elicit expert and “insider” perspectives on relevant content and workflow factors, while allowing a high degree of flexibility to capture unanticipated key issues for consideration in tool development or implementation. Documentation of interviews can range from simple detailed summaries to analyzed verbatim transcripts as is typical of rigorous qualitative research, depending on the academic versus pragmatic goals of the project.

The outcome of the literature review and key stakeholder interviews is a summary document used to drive the development of workshop materials and activities (eg, draft user profiles, value propositions, draft tool content, workflow maps, etc) and contribute material for academic manuscript development. Furthermore, results from these activities may inform the focus for site observation sessions as described below.

#### Site Observations and Workflow Analysis

UCD requires a deep understanding of workflow and the roles, responsibilities, and documents or data related to the tool in development [37,38]. All activities in the concept generation phase inform this understanding but typically site visits or observations (to correspond with key stakeholder interviews when appropriate) contribute greatly to the understanding of key issues or opportunities impacting tool building or implementation decisions. Hence, site observations are critical to a comprehensive concept generation phase. Using a structured approach adapted from evidence-based frameworks for workflow analysis in health care, such as the Workflow Elements Model and Agency for Healthcare Research and Quality’s Workflow Assessment for Health Information Technology Toolkit, qualitative and quantitative data on key elements are gathered throughout phase 1 and collected through usability testing and observations throughout the entire process [39,40].

#### Design Thinking Workshop

A design thinking workshop can happen at any point but is often a culmination of the concept generation phase, bringing together a carefully selected combination of stakeholders, including potential tool users (ideally 6-8 people) together for an extended, uninterrupted workshop (typically 4-6 hours) with an expert facilitator who guides the group through a carefully selected and sequenced body of activities designed to elicit feedback on content critical to tool development and feature specification, including exercises to create, verify, or modify (eg, user personas, opportunity statements, development exercises, value propositions, and low-fidelity prototypes of tool content or features). The design thinking workshop is key in transitioning the tool development process from the divergent ethos of the concept generation phase to the convergent cadence of the prototype development.

The types of activities conducted in a design thinking workshop vary depending on the specific needs and characteristics of an individual project, including complexity and maturity. While one project may only require 2 hours, other projects may demand an entire day’s worth of activities or multiple workshops throughout initial phases. Having representation from each of the stakeholder groups in the design workshop increases the likelihood that the resulting prototype development results in a feasible, widely acceptable tool. A typical design sprint approach in which tool development teams meet intensively for 4-5 days is rarely, if ever, feasible in the context of academic health care systems, given scheduling and logistical challenges. Maintaining the spirit of the approach and its strategies—albeit with a longer time horizon—can, from our experience, yield similar benefits [41].

#### Types of Design Thinking Workshop Activities

Workshop activities are designed to gather, explore, and refine the information needed for digital tool development related to specifying who is the target user; why they would use the tool; the context in which they will use it; and how the project team will gauge the success of the tool. From work done in the discovery phase prior to the workshop, the project team begins to develop clarity on these specifications; this includes mapping of workflows for integrating the new tool and related practices into current workstreams. For digital health service delivery products, a clear understanding of existing and new potential workflows is crucial to the design and implementation of a successful tool [42]. The products or “artifacts” of the workshop once consolidated and summarized will provide the foundation necessary for the development of an initial prototype in phase 2. Table 1 lists examples of workshop activities and their objectives.

Opportunity statement exercises are aimed at more clearly delineating facets of current practice that are not meeting needs to identify in what way new tools and processes can make measurable impact. In this type of exercise, participants are often divided into pairs or small groups and asked to provide feedback on preprepared statements and offered the chance to develop new opportunity statements. Reporting back to the entire workshop group then allows for discussion, analysis, and prioritization of statements if appropriate.

Taking a user-centered approach to health services digital tool development requires a deep understanding of not just who will be using the tool (personas and user profiles) but *how* and *when* they might use the tool to derive value. User journey mapping exercises are aimed at examining current or anticipated user experiences over time, including what user groups are doing, thinking, and feeling, and how and with what they are interacting. Insights from key informants and users gathered through interviews and within workshop activities inform the journey map, which can be created during the workshop or drafted prior to the workshop with feedback and expansion being the goal of the activity in the workshop. Journey maps are essential to the workflow analysis that is crucial to building successful HIT tools; this type of exercise and the “map” it produces provides detailed insights into role responsibilities, documents, and information content necessary for prototype development.

**Table 1:** Examples of design thinking workshop activity types.

Activity type	Objective	Example
Opportunity statement	Identify an area in which the proposed digital tool may provide value or have an impact.	In pairs, complete this statement (followed by group discussion), “How might we improve (current process/tool) so that (users) are more successful as determined by (measurable criteria)?”
Persona development	Create specific fictional users (based on the actual user research) that feature key characteristics of the anticipated user group(s).	Participants as a large group are provided with a persona worksheet for review and subsequently asked to raise and discuss, based on the key features presented in the persona story, how this should impact tool build.
User journey mapping	Examining current or anticipated user experiences over time, including what users are doing, thinking, feeling, and interacting with over time.	Facilitator presents a different user profile to each of 3 small groups, asking them to make a journey map for that user; following, each group presents their journey map for discussion and refinement.
Service blueprint	To delineate the roles and responsibilities of actors in the health care organization and potentially outside that impact, facilitate or restrict a user journey.	Facilitator presents preprepared scenario (end-to-end user journey) to map out organizational and other decisions, activities, and influencers.
Lean canvas	An actionable “business” plan to guide product development focused on problems, solutions, key metrics, and competitive advantages.	Participants shown Lean Canvas template and led through clarifying exercises regarding 9 concepts and gaps in project maturity.

While journey mapping is often referred to as a strategy for learning about the “front-stage” user experience, service blueprint exercises are geared toward uncovering the “back-stage” and “behind the scenes” organizational factors that mirror and impact those front-stage user experiences [43]. Service blueprint activities involve the diverse group of workshop participants examining, with the help of the facilitator, scenarios of user journeys to delineate the roles and responsibilities of actors in the health care organization and potentially outside that impact what happens along the user journey; particularly the ones that, in their current iteration, restrict what can and cannot be done related to the aspects of user activities and experience of interest.

The Lean Canvas is a business plan template of sorts designed to facilitate a new project’s ability to hone in on key building blocks of strategic development such as problem definition, solution, users, unique value added, and key metrics of success. A lean canvas exercise can be useful at this early stage to examine the maturity of the basic tool idea and identify gaps to be addressed for the project to have the focus and business case needed to drive successful development, implementation, and, importantly, sustained adoption [44,45].

The outcome of phase 1 is a synthesis document based on the “artifacts” (products of design activities, for example, opportunity statements, personas, and journey maps) and other findings from workshop activities. This document will drive the drafting of a prototype tool requirements document to drive prototype development and contribute further to the drafting of academic manuscripts.

### Phase 2: Prototyping and Iterative Refinement (Including Early User Testing With “Think-Aloud” Methodology)

#### Lean Startup and Agile Approaches to Digital Product Development

As a project transitions to phase 2, a tool workgroup (a group of 6-8 people pulled from the research team, representative users, key stakeholders, and members of the digital development team) is convened to solidify plans for the initial prototype and make any last tweaks to the tool or the workflow integration plan before the tool build after which the project moves to the iterative refinement phase characterized by rounds of user-testing, tool building, and implementation refinement.

In this model, as is typical in a lean startup approach, the initial prototype is refined through a multiphase, preclinical user-testing process, which serves as a clinical laboratory for building successful workflow-integrated tools with a high likelihood of adoption and adherence. Focused on the space between initial product ideation and actual building of software, lean startup as a strategy contributes a rapid, user-focused approach to idea validation with user testing [41,46,47]. In the lean approach, ideas generated by users or with the input of key stakeholders in the initial product ideation stage are validated and refined iteratively with multiple rounds of user feedback, often using prototyping with varying degrees of fidelity. If appropriate, initial user testing can occur with low fidelity (eg, paper or low-resolution wireframes) prototypes to test key assumptions before moving on to costlier and time-intensive, high-fidelity software when the tool team is more confident and committed to features and design elements to include.

Subsequent rounds of multidisciplinary workgroup sessions are interspersed with usability sessions to iteratively refine the tool, beginning with cycles of “think-aloud” usability testing sessions in which users are asked to verbalize all thoughts as they interact with the tool following a carefully scripted series of tasks of interest. The think-aloud approach is particularly well suited to exploring adoption and implementation issues [48]. Following think aloud, usability testing transitions to “near-live” testing in which users are observed carrying out representative tasks of interest with the tool during simulated clinical encounters [49-51].

Similar to the use of flight simulators for vetting new designs in the airline industry, usability testing and research is an essential part of HIT development [52]. As in aviation, clinical conditions in health care are often stressful and difficult to recreate. The lighter-weight processes for innovation in consumer digital development are frequently not sufficient in the high stakes and regulated health care environment. In addition, in HIT, there is often more than one user group; one technology may need to meet the needs of multiple clinical providers (eg, physicians, nurses, and medical assistants), as well as patients in some instances. Hence, multiple rounds of usability testing in our model reflects the unique nature of HIT compared with consumer digital development. Although data saturation is a goal, the lean philosophy takes a rapid iterative approach to user testing, which values a “good enough” level of feedback to move to the next iteration over conclusive evidence favored in traditional academic research [50]. After the tool building and implementation plan has incorporated user feedback from predeployment usability testing, the tool is ready for pilot testing in phase 3.

#### Workbook

The outcome of phase 2 is the culmination of work to date in a “workbook” designed to inform building and implementation of the tool. A workbook contains curated content and artifacts gleaned from the first 2 phases and is designed to provide a detailed, yet concise picture of the project process, as well as feature and design decisions to date and the work that informs them. This document represents an important moment in the product life cycle when project teams can use the workbook to assess gaps as well as the health and viability of the project before deciding to move on to the resource-intensive building phase. Serving as both evidence of the work to date (useful for demonstrating efforts to institutional leadership, as well as program officers, in the case of grant-funded projects), as well as a “pitch deck” for project teams to secure funding for the next phase, the workbook is a critical product in this process.

### Phase 3: Pilot Testing (“Live Usability”)

Phase 3 features pilot-testing of the tool combined with “live” usability testing prior to large-scale deployment. Pilot testing in this phase, similarly to typical research pilots, is designed to examine tool impact on workflow, uncover usability issues, and identify educational needs to be considered for inclusion by the tool workgroup before larger-scale implementation. Through the gathering and addressing of real world, *in situ* user feedback from “live” usability testing, the development team increases the likelihood that the final iteration released is likely to be acceptable and usable [53]. While it can be useful at any phase, the time-blocked binning of work in agile “sprints,” where very specific and deliberate allocation of work is binned into 2-week blocks, becomes a key characteristic of the work in phases 3 and 4.

While the Lean approach is designed to produce validated use cases and value propositions, agile techniques, such as “sprints” facilitate flexibility and efficiency, by offering strategies to support the likelihood that software will be delivered on time containing the key features that satisfy user needs [54-56]. Given the challenging environment health care poses to IT development, the lean process incorporates a sustained user-centered approach that is essential [29]. While the promotion of design thinking, prototyping, and rapid iteration is increasingly common in the health care innovation and HIT literature, coverage of these strategies tends to be superficial and isolated from the foundational principles of the lean startup and agile methodologies from which they originate.

### Phase 4: Tool Optimization, Release, and Scaling

Phase 4 focuses on ongoing training and organizational and peer support to improve acceptability and adoption of the tool postdeployment. Throughout this phase, the tool workgroup continues to meet as needed to examine and discuss tool utilization and user feedback to determine any further modifications needed to the tool itself or the implementation plan. For example, a tool built by researchers at our institution for delivering preappointment digital health assessments to patients features built-in reporting of process metrics, which are regularly reviewed by the project team in addition to ongoing user experience research for continuous improvement of tool features, functionality, and engagement.

Although additional modifications may be made to the tool itself in this later phase, our model prioritizes the role of training and organizational and peer support in the successful implementation of a digital tool [57]. Training support may consist of ongoing outreach to assess and meet training needs; organizational support may include regular contact with site leaders to assess implementation and engage in ongoing optimization to the evolving workflows; peer support may be facilitated through identification of high-volume users of the system and engaging them as implementation champions at their site.

## Discussion

### Principal Findings

A rigorous process for UCD and implementation of HIT is critical to supporting digital innovation and contributing to evidence-based medicine. Our experience developing and refining this process through multiple clinical decision support and other HIT projects yields a unique model for design in health care that, while particularly well suited to HIT development, applies to nondigital innovation as well. While design thinking and user-centered approaches are referred to with increasing frequency in the academic literature, few explicit models for HIT development exist that foster a holistic understanding to apply to both clinician- and patient-facing tools [23,58,59]. Given the value placed on holistic understanding of roles and workflows involved in the design and implementation of a new tool, future research will examine how the systematic approach put forth in the model lends itself to generating evidence to support design and implementation of HIT tools generally. High-quality user research, usability evaluation, and implementation pilot research offer value to the HIT community as a whole.

While existing models espouse the importance of design thinking, prototyping, and rapid cycles of iterative feedback, fidelity to the principles and practices of lean and agile approaches to digital development from which they came is not evident [17]. Similarly, the crucial role of usability testing both pre- and posttool deployment is not specified or emphasized. Given the complexities of health care roles and workflows, successful implementation necessitates rigorous usability testing pre- and postdeployment to truly grasp a health care user journey [48,53,60]. While recognizing the centrality, first and foremost, of the user perspective and experience and deep knowledge and consideration of the ways in which health care professionals and patients, as humans, interact with digital tools, this model incorporates strategies that also address the need for digital clinical delivery tools to incorporate the business goals and processes of the academic health system for diffusion and sustainability.

### Conclusion

A result of experience and reflection, this model is a comprehensive approach to digital tool development and implementation that promotes UCD and development, while being uniquely equipped to account for and mediate the challenges and tensions posed by the complex, highly regulated, and high stakes health care environment and the need in academic medicine to be first and foremost evidence-based. As the culmination of diverse innovation projects, this process model for user-centered digital development represents a multiphased, high-fidelity process for making HIT and other types of innovation more creative, flexible, efficient, and effective. This model is a critical step in building a rigorous approach to HIT design that incorporates a multidisciplinary, pragmatic perspective, combined with academic research practices and cutting-edge approaches to digital product development to meet the unique needs of health care.

## Abbreviations

HIT: health information technology
UCD: user-centered design
